# Does partial coating with titanium improve the radiographic fusion rate of empty PEEK cages in cervical spine surgery? A comparative analysis of clinical data

**DOI:** 10.1186/s13037-017-0127-z

**Published:** 2017-04-28

**Authors:** Andreas Kotsias, Sven Mularski, Björn Kühn, Michael Hanna, Olaf Suess

**Affiliations:** 1 Department of Orthopaedics and Traumatology, Helios Klinikum Emil von Behring, Berlin, Germany; 2 Department of Neurosurgery, Charité University Hospital, Berlin, Germany; 3 Spine and Neurotrauma Center, DRK Kliniken Westend, Spandauer Damm 130, 14050 Berlin, Germany; 4 Mercury Spine Healthcare Consulting, New York, NY USA

**Keywords:** Radiculopathy, Myelopathy, Anterior cervical diskectomy and fusion (ACDF), Polyetheretherketone (PEEK), Titanium coating, Bony fusion, Radiographic fusion, Clinical trial

## Abstract

**Background:**

Anterior cervical diskectomy and fusion (ACDF) is a well-established surgical treatment. Several types of intervertebral spacers can be used, but there is increasing evidence that PEEK cages yield insufficient fusion and thus less clinical improvement. The study aim was to assess the outcomes of single-level ACDF with an empty PEEK cage partially coated with titanium.

**Methods:**

This prospective multicenter single-arm clinical study collected follow-up data at 6, 12, and 18 months. A *post hoc* comparison was made to closely matched patients from another similar trial treated with identically designed, empty, uncoated PEEK cages.

**Results:**

There were 49 of 50 patients (98%) who met the MCID of 3+ points of improvement on VAS pain or had an 18-month VAS ≤ 1. Yet even by 18 months post-op, only 40 of 50 (80%) PEEK + Ti patients achieved complete bony fusion. The PEEK + Ti group (*n* = 49) seemed to have somewhat better fusion scores and significantly better pain relief at 6 M than the matched controls (*n* = 49), but these differences did not persist at 12 M or 18 M. Patients (with either implant) who achieved complete bony fusion had significantly better improvement of pain at 6 M and disability at 6 M and 12 M than patients that remained unfused.

**Conclusions:**

ACDF is effective treatment for cervical myelopathy and radiculopathy. Although this and other studies show that titanium fuses better, partial coating of a PEEK cage does not improve the fusion rate sufficiently or confer other lasting clinical benefit. PEEK cages fully coated with titanium should be tested in prospective randomized comparative trials.

**Trial registration:**

Prospective, multicenter, single-arm clinical observational study without an individual Trial registration number. Study design and post hoc data analysis according to the “PIERCE-PEEK study”, ISRCTN42774128, retrospectively registered 14 April 2009.

**Electronic supplementary material:**

The online version of this article (doi:10.1186/s13037-017-0127-z) contains supplementary material, which is available to authorized users.

## Background

Anterior cervical diskectomy and fusion (ACDF) is a widely used, effective treatment for radiculopathy [1–3] and myelopathy [1, 4, 5]. A recent metaanalysis of the level-one evidence on just the ACDF control arms from four randomized controlled trials reported an overall clinical success rate (according to FDA trial criteria) of 70.8% for ACDF [6]. The cages implanted in ACDF can be made of various materials, including most commonly carbon fiber, titanium, or polyetheretherketone (PEEK). PEEK has become a popular choice of material for these cages because it possesses several advantageous properties, including: 1) high durability and biocompatibility [7], 2) radiolucency, which enables clear visibility of imaging [8–13], and 3) an elastic modulus close to that of bone [8, 13], which may reduce subsidence [14–16].

But the problem with PEEK is that bony fusion of the vertebrae occurs too slowly and remains incomplete too often when ACDF is performed with empty PEEK cages without additional instrumentation [14, 17–20]. Several preclinical animal studies have also documented this issue and provided various speculative explanations for it, including the inertness of PEEK as a material, difficulty of bony ongrowth to the implant, and/or a possible immune reaction to PEEK [7, 21, 22]. Whatever the underlying biological explanation(s) might be, we recently reported on a large, prospective, multicenter clinical trial (the “PIERCE-PEEK” trial), which also showed that single-level empty PEEK cages had an 18-month rate of complete fusion of only 83% [23]. Moreover, consistent with one earlier study [20] but contradicting the rest of those earlier studies [14, 17–19] that suffered from important limitations, patients in our study who failed to achieve complete bony fusion of the vertebrae at any follow-up showed significantly less improvement of pain and disability at that follow-up and all subsequent follow-ups, compared to patients who had achieved complete fusion [23]. Since it has sometimes been reported that titanium has better rates of bony bridging [14] and bony fusion [24] than PEEK, it was widely thought that coating a PEEK cage with a thin layer of titanium would improve the radiographic fusion rate, while retaining the material advantages of PEEK mentioned above. But because the process of applying a titanium coating is technically difficult (especially in the implant’s central hole) and increases the cost, one of the first manufacturers to pursue this important innovation tried applying a solid coating of titanium on just the top and bottom surfaces of these cages, where they make contact with the vertebral endplates. It was hoped that this would be sufficient to improve the rate of bony fusion.

The aim of this study was to investigate the radiographic and clinical outcomes of a clinical trial performing single-level ACDF using empty PEEK cages coated with titanium on the top and bottom surfaces. This paper also reports a *post hoc* comparison of these patients to matched controls implanted with an uncoated but otherwise identical PEEK cage in an earlier similar trial. The present report completely replaces an earlier interim analysis on a subset of the patients reported previously in conference abstracts [25, 26].

## Methods

### Ethics

The PIERCE-PEEK study (patients included in the post hoc comparison analysis) was approved by the Ethics commission of the Charité University Hospital on May 22, 2006. The follow-up study for the PEEK + Ti group with an identical study design was approved by the local Institutional Review Board of DRK Kliniken Berlin. Patients were permitted to join the study only if they provided written informed consent and were considered competent to do so.

### Study design

The research was designed as a prospective, multicenter, single-arm clinical study. Enrollment took place from July 2009 until August 2012 in two hospitals in a major European city. We also decided to make a *post hoc* comparison to another group of patients, for whom similar prospective multicenter data had already been collected during the couple years prior to this study using an identically designed implant without any titanium coating, as described further below.

### Patients

Patients were eligible for enrollment if they were age 18+, had a degenerative condition between C3 and T1 with clinical signs of radiculopathy or myelopathy, and were otherwise indicated for a single-level ACDF. Exclusion criteria were typical and have been described previously [23]. All patients provided written informed consent.

### Surgery

All patients received a standard single-level anterior cervical diskectomy and fusion (ACDF) with implantation of a cage (described in detail below) by one of three surgeons at our hospitals. The cages were not filled with any kind of material. Additional instrumentation was not used. Post-operative bracing was not prescribed. Further details can be found elsewhere [27, 28].

### Implant

**Fig. 1:**
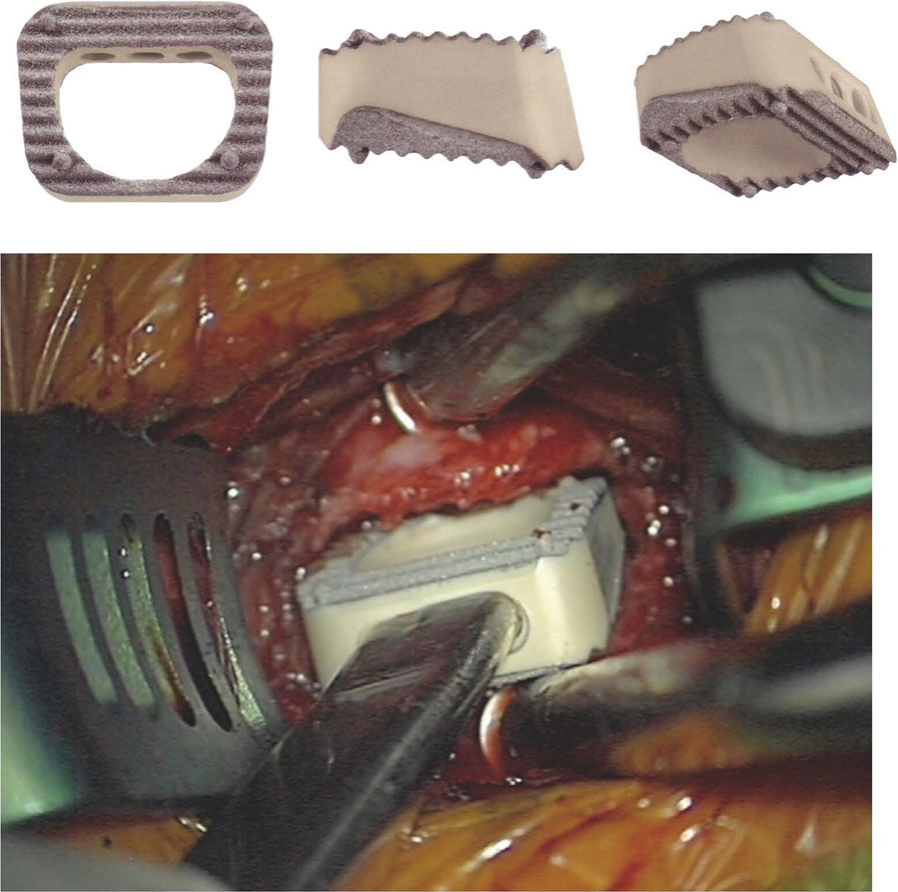
*Top*: Photographs (provided by the manufacturer) of the implant. *Left* – viewed from the *top* (notice at the four corners the tips of the tantalum pins for visualization on x-rays); *Center* – viewed laterally; *Right* – viewed obliquely to see all three planes. *Bottom*: An intraoperative photograph of the spacer being implanted into a patient, again illustrating how the base PEEK implant is coated with titanium on the *top* and bottom surfaces only

**Fig. 2:**
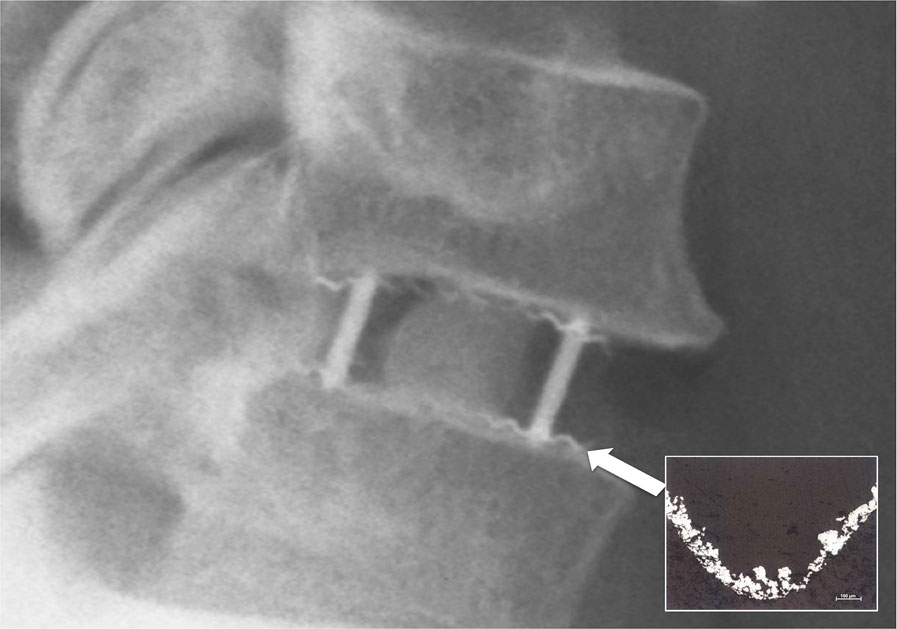
X-ray of the implant inserted between C5 and C6. As this x-ray shows, most of the implant, being made of radiolucent PEEK, cannot be seen on the x-ray. But two of the four vertical tantalum pins can be seen here (the two *vertical lines* connecting the C5 and C6 vertebral bodies); they serve to indicate the position of the implant on x-rays. Along the edge of the endplates of the vertebral bodies, we can also see two wavy lines, which are the titanium coating of the toothed *top* and *bottom* surfaces of the implant. This image thus illustrates how thin the coating of titanium is, and how it follows the form of the implant’s toothed *top* and *bottom* surfaces. The small insert is an electron-microscope image (provided by the implant manufacturer), which shows the titanium coating from another specimen of this same model of implant used in the study

All patients received a PEEK cage with a thin layer of titanium on the top and bottom surface where the cage makes contact with the vertebral bodies (“Shell” cage from Advanced Medical Technologies; Nonnweiler, Germany) (Fig. 1). The titanium layer ranged between 50 and 150 μM and had a porosity of 80%. The cages contained vertical pins of tantalum at the four corners to make the cage position visible on radiographs (Fig. 2). These cages were available to us in heights from 4 to 7 mm (in 1 mm increments) and widths of 14 to 20 mm (in 2 mm increments). Further details about this implant can be found elsewhere [27].

### Data collection

All clinical data was collected at pre-op and at 6 months, 12 months, and 18 months post-op.

### Radiographic assessment of fusion

X-rays were taken at every study timepoint and digitalized: anterior/posterior perspective of the neck in a neutral position and lateral perspective of the neck in flexion, neutral, and extension positions. The x-rays were evaluated by two independent clinicians: 1) one of two radiologists and 2) one of three neurosurgeons. In the few rare instances when the first two evaluators did not agree, the opinion of the others was sought, which always led to agreement.

Drawing on previous guidance from the US FDA and the Spinal Interbody Research Group [29–31], the evaluation of radiographic fusion was based on three criteria – bony bridging between the two vertebrae, radiolucency at the juncture of the implant and vertebra, and the amount of motion on the dynamic x-rays, as all described in greater detail previously [23], yielding a total fusion score of 0 to 7. Only patients with a score of 6–7 were considered “fused”.

### Clinical questionnaires

Pain was measured with a visual analogue scale (VAS), yielding a score of 0–10, as detailed previously [23].

Patient functioning was assessed with the Neck Disability Index (NDI), yielding a score of 0–100, as detailed previously [23].

The improvement on each of these measures at each study follow-up was calculated by subtracting the follow-up score from the pre-op score. The minimum clinically important differences (MCID) were considered to be at least 2.9 points of improvement for VAS and 20 points for NDI, as explained previously [23].

The overall outcome at 18 months post-op was assessed by the surgeon using Odom’s criteria [32].

### Comparison group

After enrollment was entirely completed, the decision was taken to make a *post hoc* comparison of these patients to a group of similar patients who had received a plain PEEK implant, in a separate but nearly identical clinical study (the “PIERCE-PEEK” study) [23]. Except for the absence of the thin titanium coating on the top and bottom surface, the PEEK cages received by the comparison group were identical in sizes, shapes, material, manufacturing, and every other aspect to the PEEK + Ti cages received by the main study group of this report. The surgeries were performed in the same way, though some of the comparison patients were operated by other surgeons at other hospitals, since this clinical study was performed only at a subset of the original study centers for uncoated PEEK cages. The surgeries in the comparison group were performed during the couple years prior to the start of the current study, but we are not aware of any relevant changes that took place between these two timeperiods that might have created a difference between these two groups. All data collection including the evaluation of the fusion was also performed in the same way. The selection of comparison patients aimed to match them for sex, age, and level, and when multiple equivalent comparators were available, also for baseline VAS and NDI, as described in more detail in Additional file 1.

### Statistical analysis

Descriptive statistics were used to characterize the patient sample. Summary statistics of radiographic and clinical outcomes were graphed. The PEEK + Ti group was compared to the PEEK only comparison group, in regards to the fusion score, the change of VAS, and the change of NDI at each of the three follow-up timepoints, as well as the Odom criteria, using Mann–Whitney rank sum tests. The odds ratio of achieving complete fusion at each follow-up timepoint, depending on which implant was used, was calculated using the McNemar mid-*p* test and a transformed Wilson score interval for the 95% CI, according to the recent recommendations and formulas from Fagerland and colleagues [33, 34]. Correlations between the fusion score at each follow-up timepoint and the improvement of VAS or NDI at that same timepoint were made with Pearson’s product moment. Comparisons were made between fused versus non-fused patients at each follow-up timepoint for the improvement of pain or functioning at the same timepoint or the Odom’s criteria, using the Mann–Whitney rank sum tests. The correlation between the fusion score at 18 months and the Odom criteria was calculated using Spearman’s rank order.

No particular *p*-value was assigned the meaning of a cutoff point between “statistically significant” versus “statistically non-significant” values. Instead, we leave it to the readers to interpret the results themselves, keeping in mind that a p-value of 0.05 (just for example) would basically mean that there is a 5% chance that the corresponding results could have been found by random chance of the study sampling and do not represent the real results that would be found in the complete population from which the study sample was drawn. Although we did always view results with *p* ≥ 0.15 as non-significant, we do not imply that results with *p* < 0.15 are necessarily significant. We recommend that readers carefully consider the meaning of each result reported, the meaning of each associated p-value, and the corresponding risks of making type I and type II errors (accepting false discoveries or rejecting true discoveries, respectively).

The software used for statistical analysis is reported in Additional file 2.

## Results

### Patients

The study sample included 27 males and 23 females. The median (range) age was 56 (22–76). The operated level was C3/C4 for 3 patients, C4/C5 for 12, C5/C6 for 19, C6/C7 for 14, and C7/T1 for 2. There was absolutely no missing data.

### Radiographic fusion

**Fig. 3:**
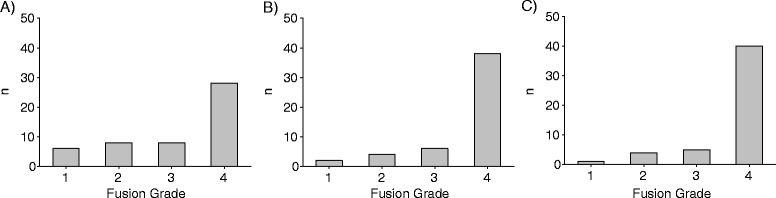
Histograms of the distribution of patients among the four grades of fusion at the three follow-up timepoints: **a** 6 months, **b** 12 months, **c** 18 months. The raw 0–7 fusion scores were grouped into four grades of fusion: grade 1 (0–2 points) was considered “no fusion”, grade 2 (3–4 points) was considered “instable ankylosis”, grade 3 (5 points) was considered “questionably stable fusion”, and grade 4 (6–7 points) was considered “complete fusion”, as previously illustrated [23]

Although the vast majority of patients achieved complete fusion by 12 and 18 months, a noteworthy minority of patients remained distributed among the other grades of radiographic fusion at all follow-ups (Fig. 3).

### Clinical outcomes

**Fig. 4:**
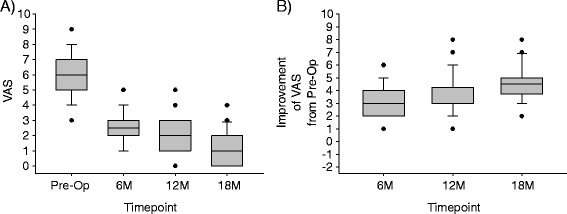
Box-and-whisker plot showing the distribution of VAS pain scores and improvement of VAS pain scores at the various study timepoints. For each box-and-whisker plot, the *middle line* of the box is the median, the *top line* of the box is the 75^th^ percentile, the *bottom line* of the box is the 25^th^ percentile, the top whisker is the 90^th^ percentile, the *bottom whisker* is the 10^th^ percentile, and the *dots* are individual patients beyond the 10^th^ and 90^th^ percentile, whereby each dot may actually be more than one dot superimposed, if two or more patients had the same score. **a** The raw VAS scores at each timepoint. **b** The improvement of the patients’ VAS scores from pre-op to the follow-up timepoint indicated. Note that an “improvement” of 0 means the follow-up score was the same as the pre-op, larger numbers indicate more improvement since pre-op, and negative numbers would mean that the patient was worse at follow-up. (For clarification, the median improvement of VAS at 12 months was 3)

Most patients had moderate pain at pre-op (Fig. 4a). The majority of patients obtained clinically important improvement of pain at all follow-ups, and no patient’s pain remained the same or worsened at any follow-up (Fig. 4b).

**Fig. 5:**
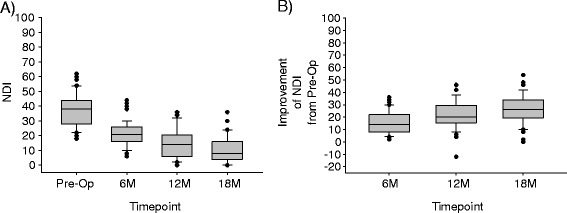
Box-and-whisker plot showing the distribution of NDI scores and improvement of NDI scores at the various study timepoints. For each box-and-whisker plot, the *middle line* of the box is the median, the *top line* of the box is the 75^th^ percentile, the *bottom line* of the box is the 25^th^ percentile, the top whisker is the 90^th^ percentile, the bottom whisker is the 10^th^ percentile, and the dots are individual patients beyond the 10^th^ and 90^th^ percentile, whereby each dot may actually be more than one *dot* superimposed, if two or more patients had the same score. **a** The raw NDI scores at each timepoint. **b** The improvement of the patients’ NDI scores from pre-op to the follow-up timepoint indicated. Note that an “improvement” of 0 means the follow-up score was the same as the pre-op, larger numbers indicate more improvement since pre-op, and negative numbers mean that the patient was worse at follow-up

Similarly, most patients had moderate disability at pre-op (Fig. 5a). The majority of patients achieved clinically important improvement of functioning at 12 and 18 months, and only one patient showed worsening of NDI at 12 months and no change from pre-op by 18 months (Fig. 5b).

The overall surgeon-rated outcome according to Odom’s criteria was “excellent” for 13 patients, “good” for 31, “fair” for 6, and “poor” for none.

There were 49 of 50 patients (98%) who either met the MCID of VAS pain improvement ≥2.9 or had an 18-month pain score ≤1. There were 41 of 50 patients (82%) who either met the MCID of NDI improvement ≥20 or had an 18-month NDI ≤10. There were 44 patients (88%) with an “excellent” or “good” Odom’s criteria. There was only one patient (2%) who did not meet any of these three aspects of success. There were 38 patients (76%) who met all three aspects.

There were no revisions or further cervical operations for any of the 50 patients within the 18 month follow-up period.

### Comparison to patients with titaniumless implants

The comparison patients were closely matched to the cases, as described in more detail in Additional file 3. Yet one case (a 22-year old male operated at C4/C5) had no plausible match among the comparison group, due to young age. This case was censored in the comparisons, leaving 49 pairs of matched cases and comparators for further analysis. There was no missing data for any of the controls.

### Comparison of radiographic fusion to patients with titaniumless implants

The fusion scores appeared somewhat better in the PEEK + Ti group than in the comparison group at 6 months, but this possible difference did not persist at 12 months and was entirely gone at 18 months (Table 1).

**Table 1:** Distribution of the fusion scores, comparing the cases to controls at each follow-up

	6 months			12 months			18 months		
	25^th^ %	Median	75^th^ %	25^th^ %	Median	75^th^ %	25^th^ %	Median	75^th^ %
Cases	4	6	6	5.5	6	6	6	6	6
Controls	3	5	6	5	6	6	6	6	6
*p*-value	0.110			0.331			0.984		

Similarly, the PEEK + Ti group was more likely to achieve complete fusion at 6 months than the control group, but this difference did not persist at 12 months and was entirely gone at 18 months (Table 2).

**Table 2:** The likelihood of achieving complete radiographic fusion, depending on the type of cervical implant: PEEK partially coated with Ti (cases) vs. uncoated PEEK (controls)

	6 months	12 months	18 months
Fused in both patients of the matched pair (*n*)	14	28	33
Fused in neither patient of the matched pair (*n*)	17	6	4
Fused only in the case of the matched pair (*n*)	13	9	6
Fused only in the control of the matched pair (*n*)	5	6	6
Odds Ratio	2.6	1.5	1.0
95% CI of the OR	0.97 – 7.00	0.56 – 4.04	0.34 – 2.94
*p*-value	0.064	0.454	>0.999

Note that the numbers (*n*) reported in the first four rows of the table are the number of *pairs* of matched patients (case + control), not of individual patients. Note also that the first two rows of the table, reporting concordant fusion status results in both patients of the matched pair, although perhaps clinically interesting, have little to no statistical relevance for comparing the two study groups. What is important instead, are the third and fourth rows, reporting discordant results of the fusion status, and the subsequent rows of statistical calculations

### Comparison of clinical outcomes to patients with titaniumless implants

The PEEK + Ti group had significantly better improvement of VAS pain scores at 6 months, but this difference did not persist at 12 months and did not clearly reappear at 18 months (Table 3).

**Table 3:** Distribution of the improvement of pain scores, comparing the cases to controls at each follow-up

	6 months			12 months			18 months		
	25^th^ %	Median	75^th^ %	25^th^ %	Median	75^th^ %	25^th^ %	Median	75^th^ %
Cases	2	3	4	3	3	4.5	3.5	5	5
Controls	2	2	3	2.5	3	4	3	4	5
*p*-value	0.029			0.394			0.121		

There was no difference between the two groups for the improvement of NDI scores at 6 or 12 months; the difference between the two groups at 18 months was probably not due to random chance but was not a clinically meaningful magnitude anyway (Table 4).

**Table 4:** Distribution of the improvement of disability scores, comparing the cases to controls at each follow-up

	6 months			12 months			18 months		
	25^th^ %	Median	75^th^ %	25^th^ %	Median	75^th^ %	25^th^ %	Median	75^th^ %
Cases	8	14	22	16	20	31	20	26	34
Controls	8	12	16	14	18	22	18	24	30
*p*-value	0.223			0.163			0.071		

There was no noteworthy difference between the two groups for the overall surgeon-rated outcome according to Odom’s criteria.

### Relationship of fusion to clinical outcomes in all patients together

The fusion score correlated with the improvement of pain scores at 6 months (*r* = 0.21, *p* = 0.04) but not at 12 months (*r* = 0.05, *p* = 0.59) or 18 months (*r* = 0.11, *p* = 0.28). Patients who achieved complete fusion had greater improvement of pain scores at the 6-month follow-up, but this difference disappeared at later follow-ups (Table 5).

**Table 5:** Distribution of the improvement of pain scores at each follow-up, comparing the patients who were completely fused to patients not completely fused at the corresponding follow-up

	6 months			12 months			18 months		
	25^th^ %	Median	75^th^ %	25^th^ %	Median	75^th^ %	25^th^ %	Median	75^th^ %
Fused	2	3	4	3	3	5	4	4	5
Not Fused	2	2	3	2	3	4	3	4.5	5
*p*-value	0.012			0.206			0.365		

From a total sample of *N* = 98, the number of fused patients was 46 at 6 months, 71 at 12 months, and 78 at 18 months

The fusion score correlated with the improvement of disability scores at 6 months (*r* = 0.28, *p* = 0.006), at 12 months (*r* = 0.34, *p* < 0.001), and 18 months (*r* = 0.21, *p* = 0.04). Patients who achieved complete fusion had greater improvement of disability scores at the 6-month and 12-month follow-ups, but non-fused patients caught up by 18 months (Table 6).

**Table 6:** Distribution of the improvement of disability scores at each follow-up, comparing the patients who were completely fused to patients not completely fused at the corresponding follow-up

	6 months			12 months			18 months		
	25^th^ %	Median	75^th^ %	25^th^ %	Median	75^th^ %	25^th^ %	Median	75^th^ %
Fused	10	16	22.5	16	22	32	19.5	24	32
Not Fused	8	12	16	14	16	20	15	22	32
*p*-value	0.040			0.015			0.347		

From a total sample of *N* = 98, the number of fused patients was 46 at 6 months, 71 at 12 months, and 78 at 18 months

The fusion score at 18 months correlated with the surgeon-rated overall outcome according to Odom’s criteria (*r* = 0.39, *p* < 0.001). The distribution of overall surgeon-rated outcomes according to Odom’s criteria was better in the patients who achieved complete fusion by 18 months than those that did not (*p* = 0.015).

## Discussion

PEEK is an advantageous and popular material for the implants used in ACDF, but it appears to sometimes cause slow or incomplete bony fusion [7, 8, 13, 14, 20, 24]. Our previous research also found many patients implanted with empty stand-alone PEEK cages did not achieve radiographic fusion even by 18 months, and patients who did not achieve fusion did not obtain as much clinical benefit as those who did [23].

Before discussing the results, it is particularly important to understand this study’s limitations and strengths. First, it must be kept in mind that this study only makes a matched comparison between two separate prospective cohorts; it is not a randomized controlled trial. Consequently, the titanium coating on the PEEK implants cannot be considered the definitive cause of any difference or lack of differences between the two groups. The comparison group was mostly treated in earlier years, sometimes by other surgeons at other hospitals, so there might be other subtle but relevant difference between the two study groups and their treatment besides the titanium coating. Second, this study only compared 49 cases and 49 controls, so it was often somewhat underpowered. For this reason, true differences between the two study groups may not be apparent or may not have strong statistical significance. If readers view the *p*-values leniently, it is often possible to see what difference titanium makes, but readers should be careful about rejecting findings here that do not quite reach a traditional level of *p* < 0.05, because this study was sometimes not quite as large as it should have been to reach that level. With these two design limitations in mind, it should be noticed that the quality of this study is otherwise high: it is a prospective multicenter study, with no missing data, and the comparison group was very well matched on sex, age, and cervical level. Yet it must be emphasized that this report is not a “case–control study” in the terminological meaning of that kind of study design. Rather, this study is a prospective single-arm cohort clinical trial, with a post hoc statistical comparison to a closely matched subsample of patients from another nearly identical prospective single-arm cohort clinical trial.

The main outcome of this study is that ACDF with this implant (a PEEK cage coated with titanium on the top and bottom surfaces only) yields very good clinical outcomes. Almost every patient obtained clinically important relief of neck pain, and about 3 in 4 patients achieved all three clinical criteria of success. However, the radiographic outcomes for this cage remain less than desirable, implying that some patients did not obtain as much benefit from ACDF with this cage as perhaps they could have.

The comparison of these two cohorts suggests that although titanium is not irrelevant, coating only the top and bottom surface remains inadequate. The PEEK + Ti group was about 2.6 times as likely to achieve fusion at 6 months than the plain PEEK group, but this difference disappeared at later timepoints. Correspondingly, the PEEK + Ti group had more improvement of pain at 6 months but not later.

This lack of lasting advantage in the PEEK + Ti group is not surprising, considering that the lateral walls of the cage – both internally and externally – remain uncoated, and thus no different from the plain PEEK cage. Perhaps partial coating of the implant endplates improves the bony ongrowth at those junctures, (though that cannot be seen on the x-rays or tested functionally in human patients). That might explain the better fusion rate at 6 months: bony ongrowth at the endplates might reduce motion on dynamic x-rays sooner. But measurable radiographic fusion requires bridging bony growth between the two vertebrae, across the implant, both around its external lateral surfaces and through its interior hole. If those lateral and internal surfaces remain uncoated, then the fusion across the cage will be no better than for a plain PEEK cage. So, obviously enough, the implants should be entirely coated in titanium.

More broadly and importantly, this study supports the strong findings of our previous study (the “PIERCE-PEEK” trial [23]) that achieving bony fusion of the vertebrae is indeed important for maximizing the clinical benefit of ACDF. Patients in the present study who were fused (with either cage) had more improvement of pain at 6 months and disability at 6 and 12 months than patients who did not achieve complete fusion. Thus the general issue of how to improve the radiographic fusion is indeed relevant to patient-experienced outcomes, and not mere technological obscuritanism.

## Conclusion

In conclusion, ACDF is an effective treatment for radiculopathy and myelopathy. Achieving bony fusion of the vertebrae is important for maximizing the clinical improvement. The speed and frequency of achieving bony fusion is suboptimal with PEEK cages. Titanium coating appears useful, but coating only the top and bottom surfaces is inadequate. A large prospective clinical trial of PEEK cages coated entirely with titanium would be an important next step toward improving treatment.

## Additional files

Additional file 1: Methods: Protocol for Selecting Matching Comparison Patients. (DOC 26 kb)

Additional file 2: Methods: Software Used for the Statistical Analysis. (DOC 22 kb)

Additional file 3: Results: Outcome of Matching Comparison Patients. (DOC 22 kb)

## Abbreviations

ACDF: Anterior cervical decompression and fusion
MCID: Minimum clinically important differences
NDI: Neck Disability Index
PEEK: Polyetheretherketone
Ti: Titanium
VAS: Visual analogue scale
