# Supplementary material for: Does partial coating with titanium improve the radiographic fusion rate of empty PEEK cages in cervical spine surgery? A comparative analysis of clinical data
Source: Patient Saf Surg. 2017 Apr 28;11:13. doi: 10.1186/s13037-017-0127-z (PMC5410055; doi:10.1186/s13037-017-0127-z)
Supplement: Supplementary file 1 — Methods: Protocol for Selecting Matching Comparison Patients. (DOC 26 kb) [file 13037_2017_127_MOESM1_ESM.doc]

Additional file 1

**Methods: Protocol for Selecting Matching Comparison Patients**

For each trial case (PEEK + Ti), a matching comparison patient (PEEK without Ti) will be selected from the main clinical trial. Comparison patients will be selected according the following rules and criteria, applied in the order listed.

1. No comparison patient will be selected more than once for inclusion into the analysis. The comparison group MUST consist of 50 unique patients.

2. The comparison patient MUST be matched for sex.

3. After matching for sex, the study aims to select a patient matched for both age and cervical level.

4a. If there is one and only such patient matched for sex, age, and level, then this is the matched comparison patient who will be included in the analysis.

4b. If there is more than one possible comparison patient matched for sex, age, and level, then the comparison patient whose baseline VAS and NDI scores appears closest to the VAS and NDI of the case patient will be selected. If there is more than one case patient with the same age, sex, and level, matched comparison patients will be chosen for each of them, simultaneously considering all available comparison patients, and all cases that need matches.

4c. The selection of comparison patients will continue according to the criteria above, until all cases have been reviewed. A note will be made of how many patients were matched exactly for sex, age, and level. Only thereafter will the selection proceed for the remaining cases who still do not have a matched comparison patient.

5. For the remaining cases that do not have a comparison patient matched exactly for sex, age, and level, a second round of selection will be made, to try to find a comparison patient (of the same sex of course) who is very close in age and still matched for cervical level.

5a. If there are no patients matched for age and level, a comparison patient will be sought that is matched for age ±1 year and matched for the same level. If there is still no such match, a match will be sought for the same level ±2 years, then ±3 years, and so on until ±5 years. A note will be made of how many patients were matched for sex and level, and approximately for age.

5b. If at any stage of the selection, there is more than one available comparison patient who meets the current stage’s criteria of selection, the comparison patient will be chosen whose baseline VAS and NDI scores are closest to those of the case.

5c. If at any stage of the selection, there is only one comparison patient who meets the current stage’s criteria of selection for more than one case patients, the comparison patient will be assigned to the case patient whose baseline VAS and NDI scores are closest to those of the comparison.

6. For the remaining cases, a third round of selection will be made, to try to find a comparison patient (of the same sex) who is exactly or approximately matched for age, operated at an adjacent level.

6a. If there are no comparison patients available matched for sex, age ±5 years, and level, then the review will start again, looking at patients matched for sex and exact age, with surgery at the immediate adjacent levels.

6b. If there are no comparison patients available matched for sex, age, and level ±1 level, a comparison patient will be sought that is matched for age ±1 year with surgery at the immediate adjacent levels. If there is still no such match, a match will be sought for the same level ±2 years, then ±3 years, and so on until ±5 years.

6c. If at any stage of the selection, there is more than one available comparison patient who meets the current stage’s criteria of selection, the comparison patient will be chosen whose baseline VAS and NDI scores are closest to those of the case.

6d. If at any stage of the selection, there is only one comparison patient who meets the current stage’s criteria of selection for more than one case patients, the comparison patient will be assigned to the case patient whose baseline VAS and NDI scores are closest to those of the comparison.

7. If there is still a case with no comparison patient matched for sex, age ±5 years, and level ±1 level, the case will be reviewed with the surgeons, and probably removed from the analysis as unmatchable.
