# Supplementary material for: Does partial coating with titanium improve the radiographic fusion rate of empty PEEK cages in cervical spine surgery? A comparative analysis of clinical data
Source: Patient Saf Surg. 2017 Apr 28;11:13. doi: 10.1186/s13037-017-0127-z (PMC5410055; doi:10.1186/s13037-017-0127-z)
Supplement: Supplementary file 2 — Methods: Software Used for the Statistical Analysis. (DOC 22 kb) [file 13037_2017_127_MOESM2_ESM.doc]

Additional file 2

**Methods: Software Used for the Statistical Analysis**

All statistical analysis and graphing was performed in SigmaPlot 11.0 (Systat Software; San Jose, CA, USA), except the odds ratio was initially calculated in OpenEpi 3.03a (Dean AG, et al; Atlanta, GA, USA), the corresponding McNemar mid-*p* test p-value was calculated in Excel for Mac 2011 v.14.6.0 (Microsoft; Redmond, WA) using the formula in the supplemental file 1 from Fagerland and colleagues [33], and the 95% CI of the OR was calculated using good old-fashion paper, pen, calculator, and human brain, according to the formulas 44 and 45 of Fagerland and colleagues [34].

33. Fagerland MW, Lydersen S, Laake P. The McNemar test for binary matched-pairs data: mid-*p* and asymptotic are better than exact conditional. BMC Med Res Methodol. 2013; 13: 91.

34. Fagerland MW, Lydersen S, Laake P. Recommended tests and confidence intervals for paired binomial proportions. Stat Med. 2014; 33: 2850-2875.
