# Supplementary material for: Does partial coating with titanium improve the radiographic fusion rate of empty PEEK cages in cervical spine surgery? A comparative analysis of clinical data
Source: Patient Saf Surg. 2017 Apr 28;11:13. doi: 10.1186/s13037-017-0127-z (PMC5410055; doi:10.1186/s13037-017-0127-z)
Supplement: Supplementary file 3 — Results: Outcome of Matching Comparison Patients. (DOC 22 kb) [file 13037_2017_127_MOESM3_ESM.doc]

Additional file 3

**Results: Outcome of Matching Comparison Patients**

Twenty-two (22) cases had comparators matched exactly for sex, age, and level.

Twenty-two (22) cases had comparators matched exactly for sex and level, and closely for age (within ± 5 years).

Three cases had comparators matched exactly for sex and age, and were operated at an adjacent level.

Two cases had comparators matched exactly for sex, were operated at adjacent levels, and were very close for age (± 1 year, which is also essentially the same age, given the data’s degree of precision).

One case (M, 22, C4/C5) had no comparable comparison patient. (The closest available comparators were two M 33 C4/C5 patients, but they have both had pre-op VAS and ODI scores double or more of the case. There were no male comparison patients under age 30 operated at C3/C4 or C5/C6, and there were no female C4/C5 comparison patients under age 30.) This unmatched case was censored from the comparative analysis.
